# Josephson diode effect from Cooper pair momentum in a topological semimetal

**DOI:** 10.1038/s41567-022-01699-5

**Published:** 2022-08-15

**Authors:** Banabir Pal, Anirban Chakraborty, Pranava K. Sivakumar, Margarita Davydova, Ajesh K. Gopi, Avanindra K. Pandeya, Jonas A. Krieger, Yang Zhang, Mihir Date, Sailong Ju, Noah Yuan, Niels B. M. Schröter, Liang Fu, Stuart S. P. Parkin

**Affiliations:** 1 Max Planck Institute of Microstructure Physics, Halle (Saale), Germany; 2 Department of Physics, Massachusetts Institute of Technology, Cambridge, MA USA; 3 Swiss Light Source, Paul Scherrer Institute, Villigen PSI, Switzerland

**Keywords:** Superconducting properties and materials, Superconducting devices

## Abstract

Cooper pairs in non-centrosymmetric superconductors can acquire finite centre-of-mass momentum in the presence of an external magnetic field. Recent theory predicts that such finite-momentum pairing can lead to an asymmetric critical current, where a dissipationless supercurrent can flow along one direction but not in the opposite one. Here we report the discovery of a giant Josephson diode effect in Josephson junctions formed from a type-II Dirac semimetal, NiTe_2_. A distinguishing feature is that the asymmetry in the critical current depends sensitively on the magnitude and direction of an applied magnetic field and achieves its maximum value when the magnetic field is perpendicular to the current and is of the order of just 10 mT. Moreover, the asymmetry changes sign several times with an increasing field. These characteristic features are accounted for by a model based on finite-momentum Cooper pairing that largely originates from the Zeeman shift of spin-helical topological surface states. The finite pairing momentum is further established, and its value determined, from the evolution of the interference pattern under an in-plane magnetic field. The observed giant magnitude of the asymmetry in critical current and the clear exposition of its underlying mechanism paves the way to build novel superconducting computing devices using the Josephson diode effect.

## Abstract (web summary)

A diode effect—asymmetric transport depending on its direction—is shown in the proximity-induced superconducting state of a Dirac semimetal.

## Main

Semiconductor junctions, which exhibit direction-dependent non-reciprocal responses, are essential to modern-day electronics^1–3^. On the other hand, a key component of many quantum technologies is the superconducting Josephson junction (JJ) where two superconductors are coupled via a weak link^4^. JJs can be used for the quantum sensing of small magnetic fields^5,6^, single-photon detection^7–9^ and quantum computation^10–12^. Despite the longstanding research on superconductivity, the realization of the superconducting analogue of the diode effect, that is, the dissipationless flow of supercurrent along one direction but not the other, has been reported only recently in superconducting thin films^13^ and JJs^14,15^. However, a clear experimental evidence for a specific mechanism leading to this effect is lacking. Recent theoretical work^16–18^ has proposed that in two-dimensional (2D) superconductors with strong spin–orbit coupling under an in-plane magnetic field, Cooper pairs can acquire a finite momentum and give rise to a diode effect, where the direction of the Cooper pair momentum determines the polarity of the effect. At the same time, although there exist prior theoretical proposals^19–22^, none of them have been experimentally realized, and a theoretical description for a field-induced diode effect in JJs (the Josephson diode effect (JDE)) that would match experimental observations has not yet been formulated.

In this work, we report the discovery of a giant JDE in a JJ in which a type-II Dirac semimetal NiTe_2_ couples two superconducting electrodes and provide clear evidence of its interrelation with the presence of finite-momentum Cooper pairing. The effect depends sensitively on the presence of a small in-plane magnetic field. Non-reciprocity Δ*I*_c_—the difference between the critical currents for opposite current directions—is antisymmetric under an applied in-plane magnetic field *B*_ip_. Further, Δ*I*_c_ strongly depends on the angle between *B*_ip_ and the current direction and is the largest when *B*_ip_ is perpendicular to the current and vanishes when *B*_ip_ is parallel to it. Moreover, we also observe multiple sign reversals in Δ*I*_c_ when the magnitude of *B*_ip_ is varied. Our phenomenological theory shows that the presence of a finite Cooper pair momentum (FCPM) and a non-sinusoidal current–phase relation account for all the salient features of the observed JDE, including the angular, temperature and magnetic-field dependences of the observed Δ*I*_c_. The distinct evolution of the interference pattern under the in-plane magnetic field further establishes the presence of FCPM in this system. We examine the plausibility of the FCPM resulting from the momentum shift of the topological surface states of NiTe_2_ under an in-plane magnetic field, by performing angle-resolved photoelectron spectroscopy (ARPES) measurements and comparison with density functional theory (DFT) calculations. This paper presents the temperature, field and angle dependences of the JDE, and is the only work to date, to the best of our knowledge, in which the fundamental properties of the effect can be explained within a single model providing clear evidence that the features are consistent with FCPM in the presence of a magnetic field.

**Fig. 1:**
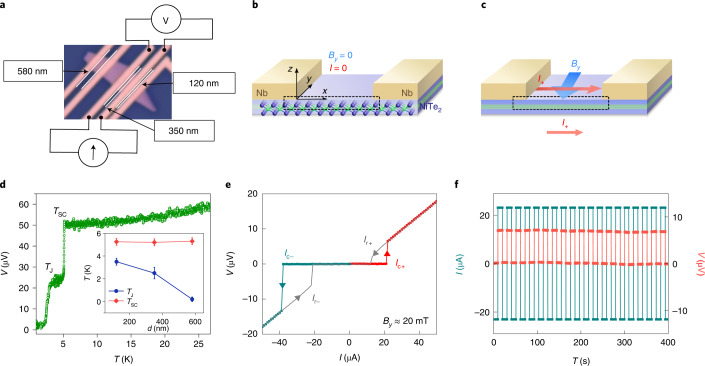
NiTe_2_ JJ device and the observation of JDE. **a**, Optical microscopy image of several JJ devices formed on a single NiTe_2_ exfoliated flake. Evidently, the edge-to-edge spacing *d* between the superconducting electrodes varies for each device. **b**, Schematic of the Josephson device. **c**, When the Josephson current flows through the junction (*I*_+_ corresponds to the Josephson current flowing in the +*x* direction), the critical current is different depending on its direction. **d**, Voltage as a function of temperature at zero field for a JJ device with *d* = 350 nm that shows two transitions at *T*_SC_ and *T*_J_. The inset shows the variations in *T*_SC_ and *T*_J_ with *d*. **e**, *I*–*V* curve of a JJ device with *d* = 350 nm at 20 mK and an in-plane magnetic field *B*_*y*_ = 20 mT showing large non-reciprocal critical current, namely, *I*_c–_ and *I*_c+_. **f**, Rectification effect observed using currents between |*I*_c–_| and *I*_c+_ for the same JJ device at 20 mK and *B*_*y*_ = 20 mT. Source data

NiTe_2_ crystallizes in a CdI_2_-type trigonal crystal structure with the space group *P*${\bar 3}$*m*1, which is centrosymmetric^23,24^ (Supplementary Section I). This 2D van der Waals material is a type-II Dirac semimetal that hosts several spin-helical topological surface states^23,24^. As discussed later, these surface states play a key role in the JDE. JJ devices were fabricated on NiTe_2_ flakes that were first mechanically exfoliated from a single crystal (Methods). Figure 1a shows the optical images of several JJ devices formed on a single NiTe_2_ flake, where the edge-to-edge separation (*d*) between the superconducting contacts (formed from 2 nm Ti/30 nm Nb/20 nm Au) in each device is different. A schematic of the JJ device is shown in the absence (Fig. 1b) and presence (Fig. 1c) of a Josephson current, where *x* is parallel to the current direction and *z* is the out-of-plane direction. The temperature dependence of the resistance of the device with *d* = 350 nm (Fig. 1d) shows two transitions: the first transition (*T*_SC_) at *T* ≈ 5.3 K is related to the superconducting electrodes^25^. A second transition (*T*_J_) takes place at a lower temperature when the device enters the Josephson transport regime such that a supercurrent flows through the NiTe_2_ layer. The dependence of both *T*_SC_ and *T*_J_ as a function of the edge-to-edge separation *d* between the electrodes is shown in Fig. 1d, inset. Although *T*_SC_ is independent of *d*, *T*_J_ decreases with increasing *d*, which corroborates that *T*_J_ corresponds to the superconducting proximity transition of the JJ device^26^.

To observe the JDE (Fig. 1b,c), we carried out current versus voltage (*I*–*V*) measurements as a function of temperature and magnetic field. Figure 1e shows *I*–*V* curves in the presence of an in-plane magnetic field *B*_*y*_ ≈ 20 mT perpendicular to the direction of the current for the device with *d* = 350 nm. The device exhibits four different values of critical current with a large hysteresis, indicating that the JJs are in the underdamped regime^27^. During the negative-to-positive current sweep (from −50 to +50 µA), the device shows two critical currents, namely, *I*_r–_ and *I*_c+_, whereas during a positive-to-negative current sweep (from +50 to −50 µA), the device exhibits two other critical currents, namely, *I*_r+_ and *I*_c–_. In the rest of the paper, we concern ourselves with the behaviour of the critical currents *I*_c–_ and *I*_c+_, which correspond to the critical values of the supercurrent when the system is still superconducting. For small magnetic fields, we find that the absolute magnitude of *I*_c–_ is clearly much larger than that of *I*_c+_ (Fig. 1e) (Supplementary Section II details the zero-field data where *I*_c+_ = |*I*_c–_|). These different values of *I*_c+_ and |*I*_c–_| mean that when the absolute value of the applied current lies between *I*_c+_ and |*I*_c–_|, the system behaves as a superconductor for the current along one direction whereas a normal dissipative metal for the current along the opposite direction. We use this difference to demonstrate a clear rectification effect (Fig. 1f) that occurs for currents larger than *I*_c+_ but smaller than |*I*_c–_|.

**Fig. 2:**
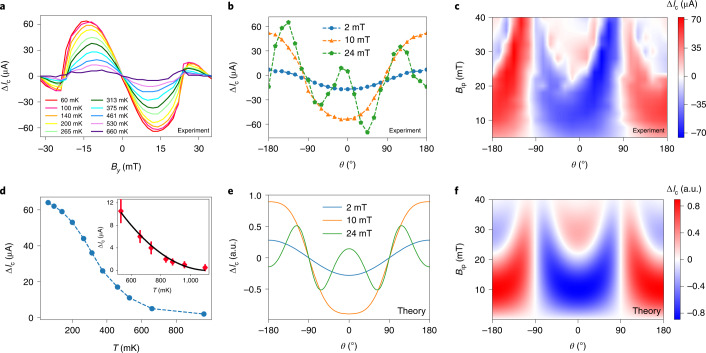
Dependence of Δ*I*_c_ on in-plane magnetic field, angle and temperature. **a**, Variation in Δ*I*_c_ as a function of *B*_*y*_ at selected temperatures in a JJ device with *d* = 350 nm. **b**,**c**, Dependence of Δ*I*_c_ as a function of in-plane magnetic field applied at different angles with respect to the current direction (**b**) and the corresponding colour contour map for the same device (**c**). Here *θ* = 0°/±180° (±90°) correspond to the in-plane magnetic field when it is perpendicular (parallel) to the current direction. **d**, Dependence of Δ*I*_c_ on temperature for *B*_*y*_ = 12 mT for the same device. The inset shows a quadratic dependence (*T* – *T*_J_)^2^ of Δ*I*_c_ on temperature close to *T*_J_. **e**,**f**, Calculation of the dependence of Δ*I*_c_ on the angle of the in-plane magnetic field (**e**) and the corresponding colour map (**f**), obtained using equation (7) for *B*_d_ = 22 mT and *B*_c_ = 45 mT. Source data

To probe the origin of the JDE, the evolution of Δ*I*_c_ (Δ*I*_c_ ≡ *I*_c+_ – |*I*_c–_|) was examined as a function of the applied in-plane magnetic field at various temperatures and angles with respect to the current direction. The dependence of Δ*I*_c_ on *B*_*y*_ (field parallel to the *y* axis and perpendicular to the current) at different temperatures demonstrates that Δ*I*_c_ is antisymmetric with respect to *B*_*y*_ (Fig. 2a). At 60 mK, Δ*I*_c_ exhibits the maximum and minimum values at *B*_*y*_ = ∓12 mT, respectively (Fig. 2a), and the ratio $\frac{{{{\Delta }}I_{\mathrm{c}}}}{{ < I_{\mathrm{c}} > }}$ is as large as 60%, where ${ < I_{\mathrm{c}} > } = \frac{{\left( {I_{{\mathrm{c}} + } + \left| {I_{{\mathrm{c}} - }} \right|} \right)}}{2}$. We find that the magnitude of $\frac{{{{\Delta }}I_{\mathrm{c}}}}{{ < I_{\mathrm{c}} > }}$ systematically increases as the distance (*d*) between the superconducting electrodes is decreased: for the device with *d* = 120 nm, $\frac{{{{\Delta }}I_{\mathrm{c}}}}{{ < I_{\mathrm{c}} > }}$ is as large as 80% (Supplementary Section III). Such a large magnitude of $\frac{{{{\Delta }}I_{\mathrm{c}}}}{{ < I_{\mathrm{c}} > }}$ at a low magnetic field (*B*_*y*_ ≈ 12 mT) makes this system unique, compared with previous reports where either the magnitude of $\frac{{{{\Delta }}I_{\mathrm{c}}}}{{ < I_{\mathrm{c}} > }}$ was found to be small or a large magnetic field was required to observe a substantial effect^13–15^. We also observe multiple sign reversals in Δ*I*_c_ when *B*_ip_ is increased (Supplementary Section IV), a previously unobserved but interesting dependence that is critical to unravelling the origin of the JDE, as discussed below.

The dependence of Δ*I*_c_ on the direction of the in-plane magnetic field with respect to the current is shown for several field strengths (Fig. 2b,c). At smaller fields, |Δ*I*_c_| is the largest when the field is perpendicular to the current (*θ* = 0°/±180°, where *θ* is the in-plane angle measured with respect to the *y* axis) and vanishes when the field and current are parallel (*θ* = ±90°). With regard to the temperature dependence of Δ*I*_c_, we find that the magnitude of Δ*I*_c_ increases monotonically as the temperature is lowered (Fig. 2a). For a quantitative understanding, the temperature dependence of Δ*I*_c_ for *B*_*y*_ = 12 mT (the field at which Δ*I*_c_ takes the largest value) is shown in Fig. 2d. At temperatures near *T*_J_, the variation in Δ*I*_c_ with temperature can be well fitted by the equation Δ*I*_c_ = *α*(*T* – *T*_J_)^2^, where *α* is a fitting parameter (Fig. 2d, inset).

We propose a possible origin of the JDE as follows. At temperatures close to *T*_J_ at which superconducting correlations develop in the proximitized region^26,28,29^ and the Josephson effect emerges (Fig. 1d), the free energy *F* of our system can be expanded in powers of the superconducting order parameters of the two superconducting electrodes, namely, *Δ*_1,2_, in the proximitized regions:

1 $$F = F_0 - \gamma _1{{\varDelta }}_1^ \ast {{\varDelta }}_2 - \frac{1}{2}\gamma _2\left( {{{\varDelta }}_1^ \ast {{\varDelta }}_2} \right)^2 + {\mathrm{c.c.}} + \ldots$$

where *F*_0_ is the free energy in the absence of Josephson coupling, and *γ*_1_ and *γ*_2_ denote, respectively, the first- and second-order Cooper pair tunnelling processes across the weak link. The presence of higher harmonics account for a non-sinusoidal current–phase relation, as commonly observed in superconductor-normal metal﻿–superconductor junctions with high transmission. Importantly, in the absence of time-reversal and inversion symmetries, *γ*_1,2_ are complex numbers, which makes the critical current non-reciprocal, as shown below.

Expressing the order parameters *Δ*_1,2_ in terms of their amplitude and phase as ${{\varDelta }}_{1,2} = {{\varDelta }}{\mathrm{e}}^{{\mathrm{i}}\varphi _{1,2}}$, the free energy takes the form *F* = *F*_0_ – 2|*γ*_1_|*Δ*^2^cos*φ* – |*γ*_2_|*Δ*^4^cos(2*φ* + *δ*), where *φ* = *φ*_2_ – *φ*_1_ + arg(*γ*_1_) is effectively the phase difference between the two superconducting regions. Note that, indeed, when both time-reversal and inversion symmetries are broken, the phase-shifted JJ is realized, as observed elsewhere^30^. Here *δ* = arg(*γ*_2_) – 2arg(*γ*_1_) is the phase shift associated with the interference between the first-order (*γ*_1_) and second-order (*γ*_2_) Cooper pair tunnelling processes. The Josephson current–phase relation then includes the second harmonic as

2 $$I(\phi ) = \frac{{2\pi }}{{{{\varPhi }}_0}}\frac{{\partial F}}{{\partial \varphi }} = \frac{{4{{e}}}}{\hbar }\left\{ {{{\varDelta }}^2\left| {\gamma _1} \right|{{{\mathrm{sin}}}}\;\varphi + {{\varDelta }}^4\left| {\gamma _2} \right|{{{\mathrm{sin}}}}\;\left( {2\varphi + \delta } \right)} \right\},$$

where ${{\varPhi }}_0 = \frac{h}{{2{{e}}}}$ is the superconducting flux quantum, *h* is Planck’s constant and *ℏ* = *h*/2*π*. When *Δ*^4^|*γ*_2_| is small, the critical current of the JJ is reached near a phase difference of *φ* ≈ ±*π*/2 and equals

3 $${{{{I}}}}_{{\mathrm{c}} \pm } \approx \left| {I\left( { \pm \frac{\uppi }{2}} \right)} \right| = \frac{{4{{e}}}}{\hbar }\left\{ {{{\varDelta }}^2\left| {\gamma _1} \right| \mp {{\varDelta }}^4\left| {\gamma _2} \right|{{{\mathrm{sin}}}}\;\delta } \right\}.$$

The non-reciprocal part of the critical current is proportional to *Δ*^4^.

4 $${\varDelta}I_{\mathrm{c}} \equiv I_{{\mathrm{c}} + } - \left| {I_{{\mathrm{c}} - }} \right| = - \frac{{8{{e}}}}{\hbar }{{\varDelta }}^4\left| {\gamma _2} \right|{{{\mathrm{sin}}}}\;\delta$$

Since the pairing potential in the proximitized layer behaves as ${{\varDelta }} \propto \sqrt {1 - \frac{T}{{T_{\mathrm{J}}}}}$ (refs. ^26,28,29^), the temperature dependence of Δ*I*_c_ near *T*_J_ is then given by

5 $${{\Delta }}I_{\mathrm{c}} \propto {{\varDelta }}^4 \propto \left( {T - T_{\mathrm{J}}} \right)^2,$$

which explains our experimentally measured temperature dependence of Δ*I*_c_ (Fig. 2d, inset).

Another feature is that ﻿Δ*I*_c_ can change sign as the applied field increases (for example, near *B*_ip_ ≈ 22 mT (Fig. 2a,c)). Such a sign reversal in ﻿Δ*I*_c_ can be reproduced by including the field dependence of the order parameters ${{\varDelta }}_{1,2} \propto \sqrt {1 - \left( {\frac{{\left| {{{\bf{B}}}} \right|}}{{B_{\mathrm{c}}}}} \right)^2}$ (where *B*_c_ is the critical field in the proximitized region) and of the phase shift *δ* due to the Cooper pair momentum.

The in-plane magnetic field *B*_ip_ can induce an FCPM in the junction. We discuss the possible origins of the finite-momentum pairing: the screening current^31^ and/or the Zeeman effect on topological surface states^32–34^. We discuss both these possible origins in detail later in the text, but note that they have the same symmetry. We also note that the phenomenological theory presented here applies to a wider class of JJs made with strongly spin–orbit-coupled materials, where the Zeeman effect can lead to FCPM^33^.

In the presence of momentum shift *q*_*x*_ under *B*_*y*_, the proximitized region effectively turns into a finite-momentum superconductor. The presence of Cooper pair momentum results in a phase shift accumulated during the Cooper pair propagation across the junction: *δ* ≈ 2*q*_*x*_*d*. At small values of the field, *q*_*x*_ must be linear in *B*_*y*_ such that

6 $$\delta \approx \pi \frac{{B_y}}{{B_d}},$$

where *B*_*d*_ is a property of the junction geometry and material that can, in principle, be determined based on the specific microscopic origin of the field-induced Cooper pair momentum. As a result, Δ*I*_c_ will have the following field dependence:

7 $${{\Delta }}I_{\mathrm{c}} \propto {{\varDelta }}^4{{{\mathrm{sin}}}}\delta \propto \left[ {1 - \left( {\frac{{\left| {{{\bf{B}}}} \right|}}{{B_{\mathrm{c}}}}} \right)^2} \right]^2{{{\mathrm{sin}}}}\left( {\pi \frac{{B_y}}{{B_d}}} \right),$$

Depending on the ratio $\frac{{B_d}}{{B_{\mathrm{c}}}}$, different scenarios can be realized from this equation; for $\frac{1}{{2(n + 1)}} < \frac{{B_d}}{{B_{\mathrm{c}}}} < \frac{1}{n}$, there are *n* sign reversals in Δ*I*_c_ when the magnetic field is applied in the *y* direction.

Figure 2e,f shows the dependences of Δ*I*_c_ on the magnitude of *B*_*y*_ and the direction of the in-plane magnetic field as obtained from our phenomenological model (equation (7)), where we used *B*_c_ = 45 mT and *B*_*d*_ ≈ 22 mT (Supplementary Section V). To explain the angular direction dependence, we take into account that the non-reciprocal part of the current is proportional only to the *x* component of momentum shift *q*_*x*_ that is proportional to the *B*_*y*_ = *B*_ip_cos*θ* component of the in-plane magnetic field. In Fig. 2f, it is evident that in each domain $- \frac{\uppi }{2} + \uppi n < \theta < \frac{\uppi }{2} + \uppi n$, the sign reversal of Δ*I*_c_ occurs where the condition $\sin \left( {\uppi \frac{{B_{{\mathrm{ip}}}\cos \;\theta }}{{B_d}}} \right) = 0$ is fulfilled, which is evident in Fig. 2c. Thus, our model successfully captures the main features of the JDE, as seen in our experimental data.

**Fig. 3:**
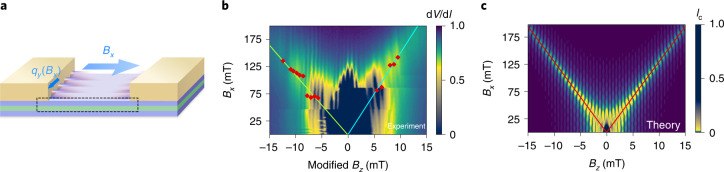
Observation of finite-momentum pairing. **a**, Schematic of the JJ in the presence of an in-plane magnetic field parallel to the current direction, leading to the emergence of an FCPM in the *y* direction. The waves illustrate the oscillations of the real part of the order parameter in the vicinity of the leads that occur due to the finite-momentum pairing. **b**, Dependence of $\frac{{{\mathrm{d}}V}}{{{\mathrm{d}}I}}$ on in-plane (*B*_*x*_) and out-of-plane (*B*_*z*_) magnetic field for a JJ device with *d* = 350 nm, showing the evolution of the interference pattern due to the applied in-plane magnetic field. The colour corresponding to the normalized magnitude of $\frac{{{\mathrm{d}}V}}{{{\mathrm{d}}I}}$ is shown on the right side of each figure. The red diamonds correspond to the positions of the centres of the peaks with respect to *B*_*x*_ and the solid lines correspond to the linear fits used to determine the slopes of the side branches (Supplementary Section VI). **c**, Calculation of the evolution of the interference pattern expressed as a function of *B*_*z*_, where the colour represents the value of *I*_c_. The solid lines mark the side branches with the slope corresponding to the average of the experimentally obtained slopes $\left( {\frac{{{{\Delta }}B_x}}{{{{\Delta }}B_z}}} \right)_{{\mathrm{avg}}}$ ≈ 13. We note that the qualitative behaviour of $\frac{{{\mathrm{d}}V}}{{{\mathrm{d}}I}}$ is the same as that of *I*_c_, including periodicity with respect to *B*_*z*_ and slope of the side branches^34^.

To confirm the emergence of a FCPM under an in-plane magnetic field, we examine the evolution of the interference pattern ($\frac{{{\mathrm{d}}V}}{{{\mathrm{d}}I}}$ versus *B*_*z*_) with the field *B*_*x*_ parallel to the current direction (Fig. 3b). This interference pattern has a similar resemblance with the Fraunhofer pattern such that a higher *I*_c_ in the latter translates to a lower $\frac{{{\mathrm{d}}V}}{{{\mathrm{d}}I}}$ in the former^33,34^. For this in-plane field orientation (*B*_*x*_), the Cooper pairs acquire finite momentum 2*q*_*y*_ along the *y* direction (Fig. 3a), which should not generate a JDE but is expected to change the interference pattern. When a Cooper pair tunnels from position (*x* = 0, *y*_1_) in the left superconductor with order parameter ${{\varDelta }}_1\left( {y_1} \right) = {{\varDelta }}{\mathrm{e}}^{2{\mathrm{i}}q_yy_1}$ to the superconductor on the right at (*x* = *d*_eff_, *y*_2_) with ${{\varDelta }}_2\left( {y_2} \right) = {{\varDelta }}{\mathrm{e}}^{2{\mathrm{i}}q_yy_2 + {\mathrm{i}}\varphi _0}$ (where *d*_eff_ = *d* + 2*λ* is the effective length of the junction and *λ* = 140 nm is the London penetration depth in Nb (ref. ^35^)), the contribution of this trajectory to the current involves a phase factor proportional to the Cooper pair momentum^33,34^:

8 $${{\Delta }}\varphi = 2q_y(y_2 - y_1),$$

in addition to the usual phase factor $\frac{{2\uppi B_zd_{{\mathrm{eff}}}\left( {y_1 + y_2} \right)}}{{{{\varPhi }}_0}}$ due to the magnetic flux associated with *B*_*z*_. The interference pattern is a result of interference from all such trajectories (Fig. 3b,c).

Due to the additional phase Δ*φ* from the in-plane-field-induced Cooper pair momentum, the interference pattern evolves as the in-plane field is increased, splitting into two branches. The Cooper pair momentum 2*q*_*y*_ can be extracted from the slope of the side branches^33,34^ (Fig. 3b, solid lines). For the *d* = 350 nm device, we estimate the average slope to be $\frac{{B_x}}{{B_z}}$ ≈ 13 (Supplementary Section VI details the estimation process). In Fig. 3c, we show the calculated critical Josephson current, obtained by summing over quasi-classical trajectories (Supplementary Sections VII and VIII), which has a qualitatively similar behaviour to the differential resistance (Fig. 3b), with the same period of oscillations (ẟ*B*_*z*_ ≈ 0.8 mT) and the slope of the side branches. The slope of the side branches can be expressed as

9 $$\frac{{2q_y}}{{B_z}} \approx \frac{{\uppi d_{{\mathrm{eff}}}}}{{{{\varPhi }}_0}}.$$

From this and the value of the slope $\frac{{B_x}}{{B_z}}$ extracted from the experiment, we find that at *B*_*x*_ = 12 mT, the Cooper pair momentum is 2*q*_*y*_ ≈ 1.6 × 10^6^ m^–1^.

Let us compare the estimate of the Cooper pair momentum based on the evolution of the interference pattern with the results of the JDE measurements mentioned above. The maximum JDE is achieved when the phase shift *ẟ* = 2*q*_*x*_*d* equals approximately 0.5π, which corresponds to the Cooper pair momentum 2*q*_*x*_ = $\frac{\delta }{d}$ ≈ 4.5 × 10^6^ m^−1^ at *B*_*x*_ = 12 mT. Although the JDE and interference peak splitting are measured under in-plane magnetic fields along two orthogonal directions, the field-induced Cooper pair momenta 2*q*_*x*_ and 2*q*_*y*_ estimated from these measurements are of the same order of magnitude, further strengthening our conclusion that both effects arise from the FCPM.

It is remarkable that a JJ device comprising a centrosymmetric material such as NiTe_2_ exhibits a large JDE effect arising from finite-momentum pairing. There exist several possible mechanisms that could be responsible for FCPM in our experiment. One candidate is the current screening effect. This mechanism, which lies in the emergence of the finite-momentum Cooper pairing due to Meissner screening, has already been demonstrated to be able to give rise to FCPM^31^ and has been predicted to be responsible for a robust and large JDE in short junctions^36^. We estimate the value of finite-momentum Cooper pairing arising from screening in Nb contacts. The thickness of Nb leads (30 nm) is much smaller than the London penetration depth, and therefore, we estimate $q_x \approx \frac{{{e}}}{\hbar }B_y\frac{{h_{{\mathrm{Nb}}}}}{2}.$ At *B*_*y*_ = 20 mT, this corresponds to 2*q*_*x*_ ≈ 10^6^ m^–1^. Since this is smaller than the observed value of Cooper pairing, it alone cannot account for the observed effect.

**Fig. 4:**
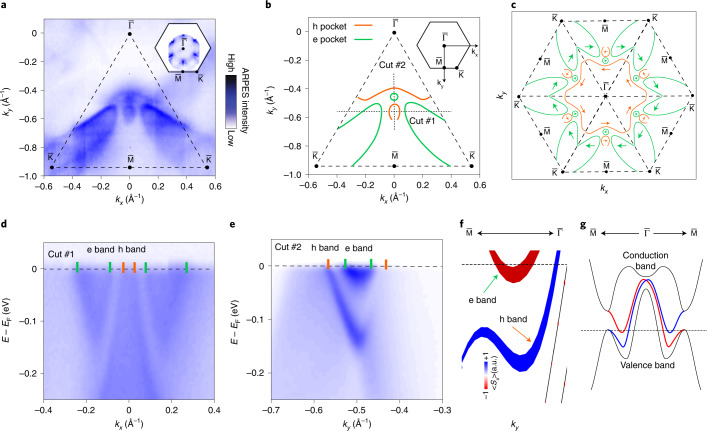
Spin-polarized surface states of NiTe_2_. **a**, Close-up of the Fermi surface measured with ARPES using photon energy *hv* = 56 eV and horizontal linear polarization. The inset shows the larger fraction of the surface Brillouin zone measured with *hv* = 25 eV and horizontal linear polarization. **b**,**c**, Schematic of the surface electron (green lines) and hole (orange lines) pockets deduced from the ARPES data. The arrows in **c** show the spin texture of the surface states. **d**,**e**, ARPES dispersion along cut #1 (**d**) and cut #2 (**e**) (the cuts are shown in **b**), showing the electron (green lines) and hole (orange lines) character of the bands. The data shown in **d** were measured with 56 eV and those in **e** with 25 eV, both with horizontal linear polarization. **f**, DFT calculations of the energy–momentum dispersion along the ${{{\bar{\mathrm \Gamma }}}} - \bar M$ direction. Two surface states are highlighted, the line width is proportional to the surface contribution and the component of the spin polarization perpendicular to the momentum is shown in colour. **g**, Schematic showing the relative position of the dispersion of the surface states along the ${{{\bar{\mathrm \Gamma }}}} - \bar M$ direction with respect to the conduction and valence bands.

Another plausible origin of the FCPM is related to the Zeeman shift of the proximitized topological surface states of NiTe_2_. Previous studies^23,24^ have reported the presence of spin-polarized surface states in NiTe_2_ that cross the Fermi level. Here we have used ARPES to examine the detailed fermiology of these surfaces states and estimate their Fermi energy and velocity (Supplementary Sections IX and X). Figure 4a displays a close-up of the experimental Fermi surface, showing both sharp surface-state bands and diffuse bulk bands that are broadened due to the limited penetration depth of the photoelectrons. The full Fermi surface in the surface Brillouin zone of NiTe_2_ is shown in Fig. 4a (inset). A schematic representing the surface electron and hole pockets (indicated by green and orange lines, respectively) is shown in Fig. 4b,c, where the arrows indicate the spin texture. The electron-like and hole-like character of the Fermi surface pockets can be examined from the linecuts (Fig. 4d,e). The energy–momentum dispersion of two topological surface states along the ${{{\bar{\mathrm \Gamma }}}} - \bar M$ direction are shown in Fig. 4e, which is qualitatively reproduced by our ab initio calculations (Fig. 4f) that also indicate their spin polarization. Note that the calculation slightly underestimates the energy at which the lower-lying surface state merges with the valence-band bulk continuum in the experiment. The origin of these surface states is the band inversion of the valence and conduction bands above the Fermi level^24,37^, which leads to the formation of a spin-helical Dirac surface state that connects the conduction and valence bands (Fig. 4g), similar to the surface state in a topological insulator. The upper branch of this Dirac surface state forms an electron pocket with a relatively small Fermi energy (*E*_F_ ≈ 15 meV) and Fermi velocity *v*_F_ ≈ 0.4 × 10^5^ m s^–1^, as well as a hole pocket with a larger Fermi velocity of 3.3 × 10^5^ m s^–1^.

The data shown in Fig. 4d–g were used for deducing the spin structure of the surface states shown in Fig. 4b,c, which is in agreement with the experimental reports^23,24,37^. The spin texture of the surface states (Fig. 4c) clearly shows one (inner) helical hole state and one (outer) helical electron state. The helicity of the hole and electron states is opposite. In the presence of an in-plane magnetic field, the Fermi surfaces of these states shift in the same direction; furthermore, in the proximity of the superconductor, finite-momentum Cooper pairing is realized. The momentum shift of these states in a magnetic field can be substantial: the surface states of topological insulators can have large *g*-factors, for example, a *g*-factor of ~60 has been found in a previous report^38^. However, because of the complexity of the surface-state structure, we leave quantifying the FCPM to a further study. However, the presented analysis suggests that there will be a finite, and likely substantial, contribution of the spin–momentum-locked surface states of NiTe_2_ to the finite-momentum Cooper pairing.

In summary, we have shown that a JJ device involving a type-II Dirac semimetal NiTe_2_ exhibits a large non-reciprocal critical current in the presence of a small in-plane magnetic field oriented perpendicular to the supercurrent. The behaviour of the critical current together with the evolution of the interference pattern under the application of an in-plane magnetic field provides compelling evidence for finite-momentum Cooper pairing. Whether or not this mechanism is generic to the family of Dirac semimetals remains an open question.

## Methods

### Exfoliation of NiTe_2_ flakes

Thin NiTe_2_ flakes were exfoliated from a high-quality NiTe_2_ single crystal (from HQ Graphene) using a standard Scotch-tape exfoliation technique and placed on a Si(100) substrate with a 280-nm-thick SiO_2_ on top. Although NiTe_2_ is known to be very stable under ambient conditions^39^, the exfoliation was carried out in a glove box under a nitrogen atmosphere, with water and oxygen levels each below 1 ppm. The thinnest flakes were identified from their optical contrast in an optical microscope and subsequently used to prepare the devices.

### Device fabrication and electrical measurements

All the devices used in our experiments were fabricated using an electron-beam-lithography-based method. Before exposure, each substrate was spin coated with an AR-P 679.03 resist at 4,000 rpm for 60 s followed by annealing at 150 °C for 60 s. After electron-beam exposure and subsequent development, contact electrodes were formed from sputter-deposited trilayers of 2 nm Ti/30 nm Nb/20 nm Au for the JJ devices. The contacts formed from 2 nm Ti/80 nm Au were deposited for the Hall bar devices.

Electrical transport measurements were performed in a Bluefors LD-400 dilution refrigerator with a base temperature of 20 mK and equipped with high-frequency electronic filters (QDevil ApS). Angle-dependent magnetic-field measurements were carried out using a 2D superconducting vector magnet integrated within the Bluefors system. A small consistent offset in the magnetic field, of the order of ~1.5 mT, was observed as the magnetic field was swept due to likely flux trapping within the superconducting magnet coils. Direct current (d.c.) voltage characteristics of the JJ devices were measured using a Keithley 6221 current source and a Keithley 2182A nanovoltmeter. Differential resistance measurements were performed using a Zurich Instruments MFLI lock-in amplifier with a multi-demodulator option using a standard low-frequency (3–28 Hz) lock-in technique. The critical currents for the Fraunhofer pattern were measured in combination with a Keithley 2636B voltage source to sweep the d.c. bias.

### ARPES experiments

To explore the momentum-resolved electronic structure, ARPES measurements were carried out on a NiTe_2_ single crystal cleaved along the [0001] direction. All the experiments were carried out at the ULTRA endstation of the SIS beamline at the Swiss Light Source, Switzerland, with a Scienta Omicron DA30L analyser. Each crystal was cleaved in situ under ultrahigh vacuum at 20 K to ensure no contamination of the surface. The base pressure of the system was better than 1 × 10^−10^ mbar.

### Calculation of evolution of interference pattern in an in-plane magnetic field

We calculate the Josephson current by considering the quasi-classical trajectories of Cooper pairs across the junction^34^ (Supplementary Fig. 12):

$$I\left( {{{\Delta }}\varphi _0,B_x,B_z} \right) = \mathop {\smallint }\limits_0^W \mathop {\smallint }\limits_0^W {\mathrm{d}}y_1{\mathrm{d}}y_2\frac{1}{{d_{{\mathrm{eff}}}^2 + \left( {y_2 - y_1} \right)^2}}\sin \left( {{{\Delta }}\varphi \left( {B_x,B_z} \right)} \right),$$

where

$${{\Delta }}\varphi \left( {B_x,B_z} \right) = {{\Delta }}\varphi _0 + \frac{{2\uppi B_zd_{{\mathrm{eff}}}\left( {y_1 + y_2} \right)}}{{{{\varPhi }}_0}} + 2q_y(B_x)\left( {y_2 - y_1} \right)$$

is the total phase difference for a trajectory that starts at (0, *y*_1_) and ends at (*d*_eff_, *y*_2_), *d*_eff_ = *d* + 2*λ* and *λ* = 140 nm is the London penetration depth^35^. Further, Δ*φ*_0_ is the phase difference between the order parameters in the two superconducting leads in the absence of an applied field. We have neglected the effect of the finite thickness of NiTe_2_.

To calculate the evolution of the interference pattern, we compute the Josephson current using the equation above and maximize it by varying Δ*φ*_0_, which allows one to find *I*_c_. To compare the theoretical and experimental predictions, we further adjust the value of the effective junction separation *d*_eff_ because of flux focusing^34^, which is carried out by calculating the Fraunhofer pattern at a zero in-plane magnetic field (giving a slightly different but still qualitatively similar dependence to $\frac{{\sin \left( \frac{{\uppi {{\varPhi }}}}{{{{\varPhi }}_0}}\right) }}{\left({{\frac{{\uppi {{\varPhi }}}}{{{{\varPhi }}_0 }}}}\right)}$) and by fitting the position of the first minimum to the experimental value. We find the linear dependence of the parameter *q*_*y*_(*B*_*x*_) and the vertical scale of the theoretical plot by using the average slope of the side branches $\frac{{B_x}}{{B_z}}$ from the experiment and matching it to the slope of the calculated pattern^34^: $\frac{{2q_y}}{{B_z}} \approx \frac{{\uppi d_{{\mathrm{eff}}}}}{{{{\varPhi }}_0}}$.

### DFT calculation of NiTe_2_ energy spectrum

For the Dirac semimetal NiTe_2_, the lattice parameters are *a* = *b* = 3.857 Å, *c* = 5.262 Å, *α* = *β* = 90° and *γ* = 120°. We first perform DFT calculations with the full-potential local-orbital program^40^. The band-structure calculation is done with a fine *k*-point mesh including up to 50 points. Since we are using atomic basis sets, a high-symmetry tight-binding Hamiltonian is constructed using the automatic Wannier projection flow^41^. The Bloch states are projected onto a small local basis set (34 bands for 3 atoms) around the Fermi level, which consists of 3*d*, 4*s* and 4*p* orbitals for Ni and 5*s* and 5*p* orbitals for Te. The projected tight-binding Hamiltonian perfectly reproduces the DFT band structures even up to 5 eV away from the Fermi level. The spin-projected surface states are calculated in a slab geometry with 10 to 20 layers from a Wannier tight-binding model.

## Online content

Any methods, additional references, Nature Research reporting summaries, source data, extended data, supplementary information, acknowledgements, peer review information; details of author contributions and competing interests; and statements of data and code availability are available at 10.1038/s41567-022-01699-5.

## Supplementary information

Supplementary Information Supplementary Sections I–X and Figs. 1–16.

## Data Availability

Source data are provided with this paper. All other data that support the findings of this study are available from the corresponding authors upon reasonable request.

## Source data

Source Data Fig. 1 All the combined line plots of Fig. 1.
Source Data Fig. 2 All the combined line plots of Fig. 2.
