# Supplementary material for: Josephson diode effect from Cooper pair momentum in a topological semimetal
Source: Nat Phys. 2022 Aug 15;18(10):1228–33. doi: 10.1038/s41567-022-01699-5 (PMC9537108; doi:10.1038/s41567-022-01699-5)
Supplement: Supplementary file 1 — Supplementary Sections I–X and Figs. 1–16. [file 41567_2022_1699_MOESM1_ESM.pdf]

nature portfolio

https://doi.org/10.1038/s41567-022-01699-5

Supplementary information

Josephson diode effect from Cooper pair momentum in a topological semimetal

In the format provided by the authors and unedited

Supplementary Information for

Josephson diode effect from Cooper pair momentum in a topological semimetal

Banabir Pal[1]*, Anirban Chakraborty[1]*, Pranava K. Sivakumar[1]*, Margarita Davydova[2]*, Ajesh K. Gopi[1], Avanindra K. Pandeya[1], Jonas A. Krieger[1], Yang Zhang[2], Mihir Date[1], Sailong Ju[3], Noah Yuan[2], Niels B.M. Schröter[1], Liang Fu[2#] and Stuart S.P. Parkin[1#]

1. Max Planck Institute of Microstructure Physics, Halle (Saale) 06120, Germany
2. Department of Physics, Massachusetts Institute of Technology, Cambridge, MA 02139, USA
3. Swiss Light Source, Paul Scherrer Institute, CH-5232 Villigen PSI, Switzerland

* Equal author contributions

\# Email: stuart.parkin@mpi-halle.mpg.de, liangfu@mit.edu

Contents:

I. Crystal structure, atomic force microscopy and other electrical characterization of $NiTe_2$
II. Characterization of Josephson junction devices at zero field
III. The dependence of ΔI_c on superconducting electrode separation and JDE effect in thick films
IV. Multiple sign reversals in ΔI_c as a function of in-plane magnetic field
V. Determination of B_c and B_d for the theoretical modeling
VI. Tilting correction and slope determination from the interference pattern to estimate finite momentum Cooper pairing
VII. Calculation of the evolution of the Fraunhofer pattern
VIII. Effect of the finite film thickness of $NiTe_2$ on the evolution of the Fraunhofer pattern
IX. ARPES data and Fermi velocity of two surface states
X. Photon energy dependent ARPES measurements for the identification of the surface states

I. Crystal structure, atomic force microscopy and other electrical characterization of $NiTe_2$

$NiTe_2$ is a layered van der Waals materials which can be easily exfoliated**.** $NiTe_2$ crystallizes in a trigonal crystal structure with the space group $P_{\bar{3}m1}$. The crystal structure is comprised of $NiTe_2$ tri-layers stacked along the c axis, each tri-layer composed of a Ni sheet sandwiched between two Te sheets. The crystal structure of $NiTe_2$ along two crystallographic directions is shown in Fig. S1a and b.

Atomic force microscopy (AFM) measurements were performed on the fabricated devices to measure both the thicknesses of the $NiTe_2$ flakes as well as to estimate the distance between the superconducting electrodes in the Josephson junction devices. A typical AFM image of a device is shown in the inset of Fig. S1c from which we estimate the $NiTe_2$ flake thickness to be ~22 nm. The distance between the superconducting electrodes for 2 typical devices are shown in the AFM images in Fig. S1d and e.

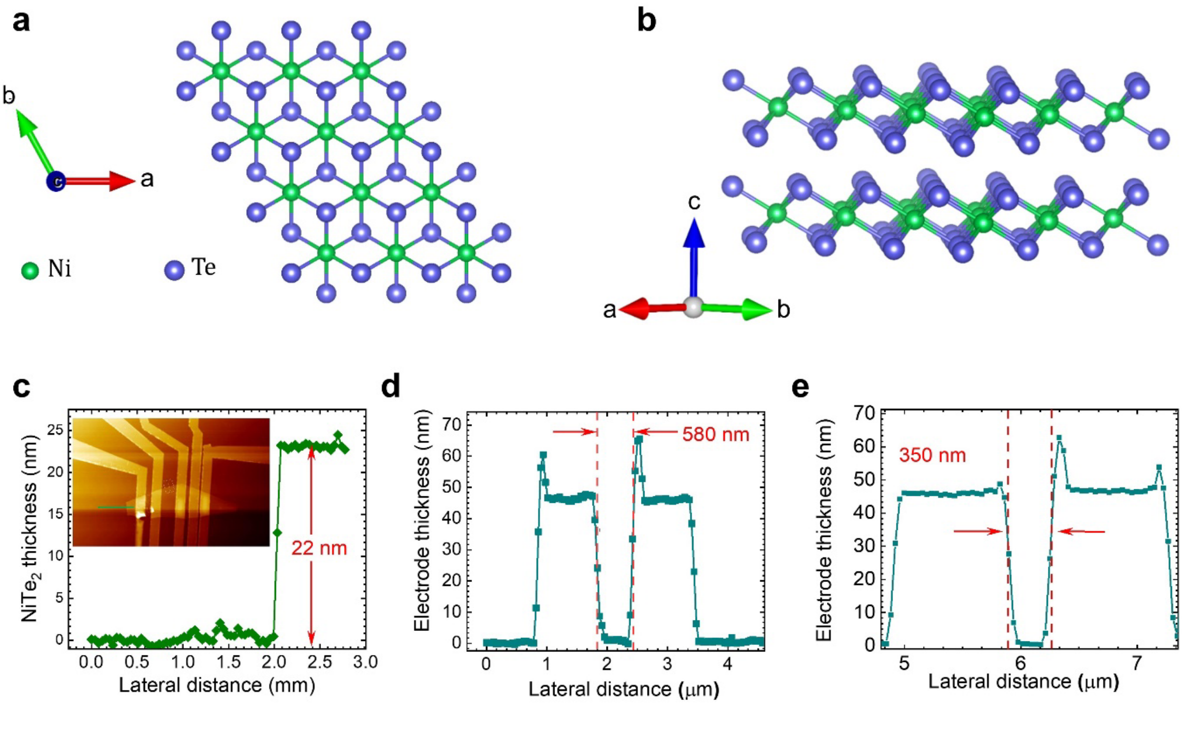

Fig. S1| Crystal structure of $NiTe_2$ and AFM of Josephson Junction devices. a, and **b,** Hexagonal crystal structure of $NiTe_2$ viewed along two crystallographic directions. Ni and Te atoms are shown in green and violet. **c**, AFM image of a $NiTe_2$ flake with an estimated thickness of ~22 nm. This flake is used for the results that are presented in the main text. **d,**

and **e,** Distance between the superconducting electrodes for 2 devices measured from an AFM image.

Transport properties of the exfoliated $NiTe_2$ flake are shown in Fig. S2. The temperature dependence of the sample resistance shows that the sample is metallic. The optical image of the flake with contacts is shown in the inset in Fig. S2a. The magnetoresistance was measured using magnetic fields of up to 14 T that were applied along various directions (Fig. S2b). A large magnetoresistance of ~ 80% is observed at 2 K when the applied field is perpendicular to the plane of the crystal. These results are consistent with previous reports[1].

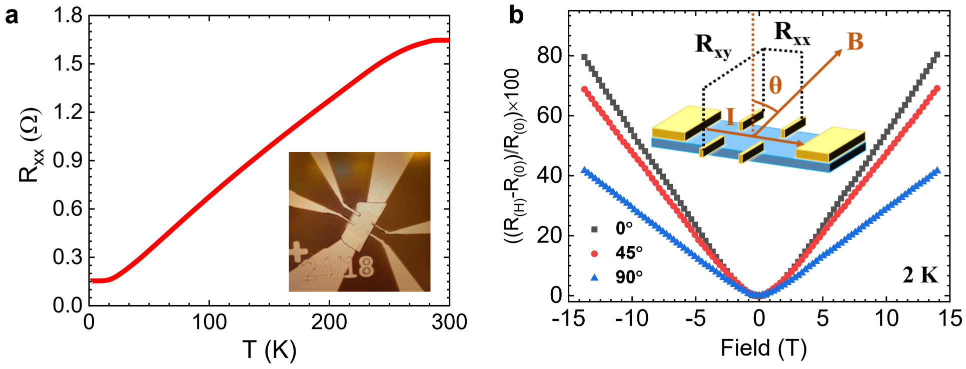

Fig. S2| Electrical measurements of $NiTe_2$ hall bar devices. a, Temperature dependence of the four-probe resistance of the $NiTe_2$ crystal. **b,** Magnetoresistance of the same device at 2 K.

II. Characterization of Josephson junction devices at zero field

Fig. S3a shows current (I) - voltage (V) curves for three Josephson junctions with superconducting electrode separations, $d = 120$, 350 and 580 nm at $T = 20$ mK, in the absence of any magnetic field. It can clearly be seen that there is no diode effect in the absence of a magnetic field. Large hysteresis in the I-V curves suggest that the devices are in the underdamped regime[2,3]. With increasing d the normal state resistance (R_n), that is determined from the slope of the I-V curve, increases (Fig. S3b) and the corresponding critical current (I_c) decreases (Fig. S3a), as expected for a proximity-induced Josephson junction. The fitting of the d dependence of the characteristic voltage ($V_c = I_c R_n$) using an exponential dependence $\sim e^{(-\frac{d}{\xi})}$ shows that the characteristic decay length (ξ) in this device is ~ 414 nm (Fig. S3c).

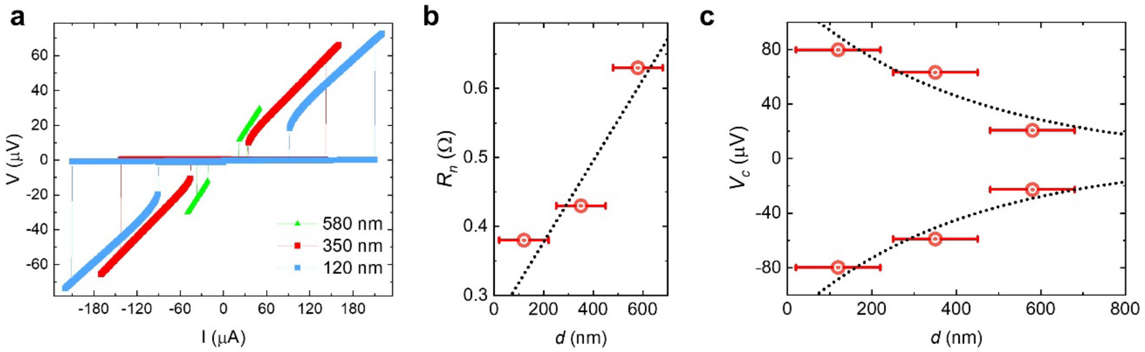

Fig. S3| Electrical characterization of Josephson junction devices. a, Current (I)-voltage (V) curves for three Josephson junctions with superconducting electrode separations, $d\ = 120$, 350 and 580 nm. **b,** Dependence of the normal state resistance (R_n) on d for three devices. **c,** Dependence of the critical voltage on d.

III. Dependence of ΔI_c on superconducting electrode separation and JDE effect in thick films

Fig. S4 (a) - (c) displays the dependence of ΔI_c on the in-plane magnetic field (perpendicular to the current direction) for three different devices with different superconducting electrode separations d = 120 nm, 350 nm and 580 nm. For each device we find that ΔI_c is antisymmetric with respect to the in-plane magnetic field and is maximum when the in-plane magnetic field is perpendicular to the current.

The magnitude of **the non-reciprocity increases as the distance between the superconducting electrodes decreases.** For the device where the spacing between the superconducting electrodes is 120 nm, the magnitude of the JDE ($\approx \frac{\Delta I_c}{<I_c>}$) is as large as 80%. We summarize our results and compare them with previous reports in Fig. S4 d on related non-reciprocal effects in Josephson junctions. We note that the magnitude of the non-reciprocity ($\approx \frac{\Delta I_c}{<I_c>}$) in our JJs is significantly higher than previous reports.

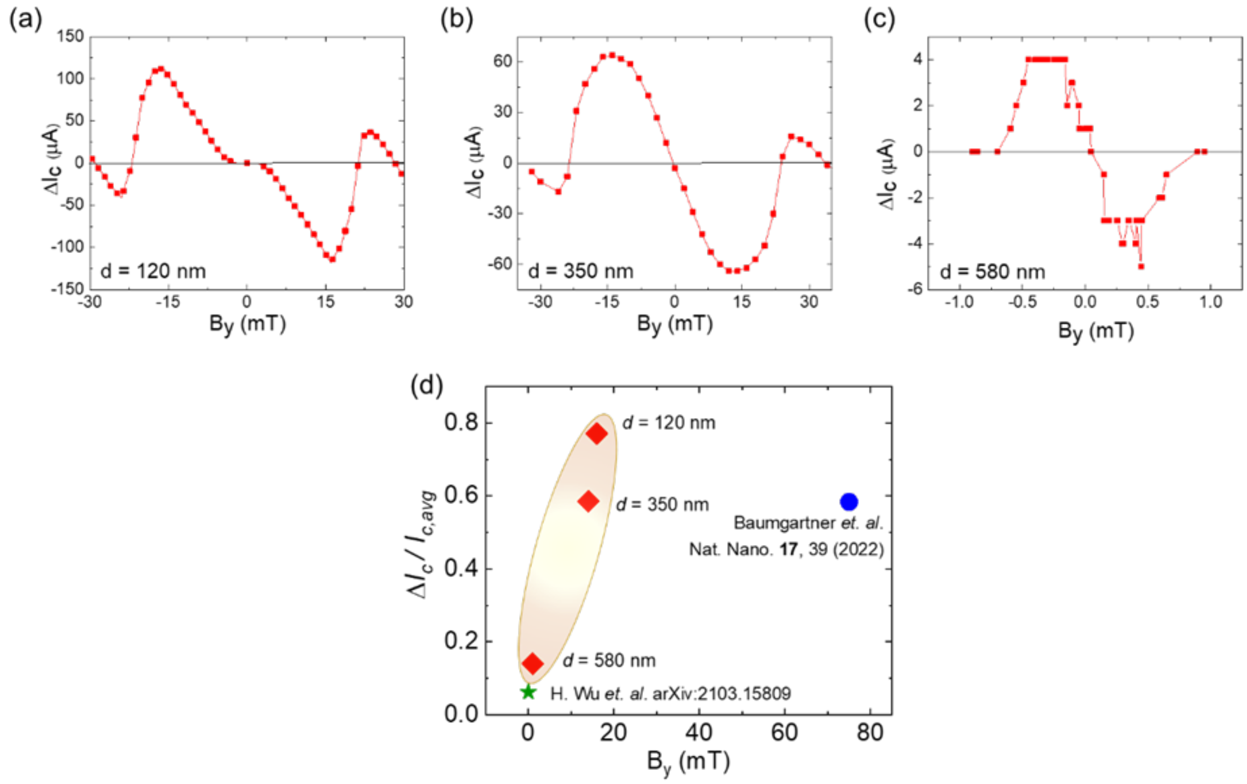

Fig. S4| Dependence of ΔI_c on superconducting electrode separation: Variation of ΔI_c on the in-plane magnetic field perpendicular to the current direction for devices with *d* = (a) 120 nm (b) 350 nm and (c) 580 nm. (d) Comparison of the JDE effect in our device with previous reports.

Non-reciprocity in a Josephson junction comprised of 60 nm thick $NiTe_2$:

The non-reciprocal effect in a Josephson junction formed from a 60 nm thick $NiTe_2$ flake is shown in Fig. S5. Fig. S5 (a) shows an optical micrograph of the junctions formed on a single $NiTe_2$ flake. This 60 nm thick sample shows a JDE effect as can be observed from the I-V curve in Fig. S5 (b) that was acquired for an in-plane magnetic field $B_y = 40\ mT$.

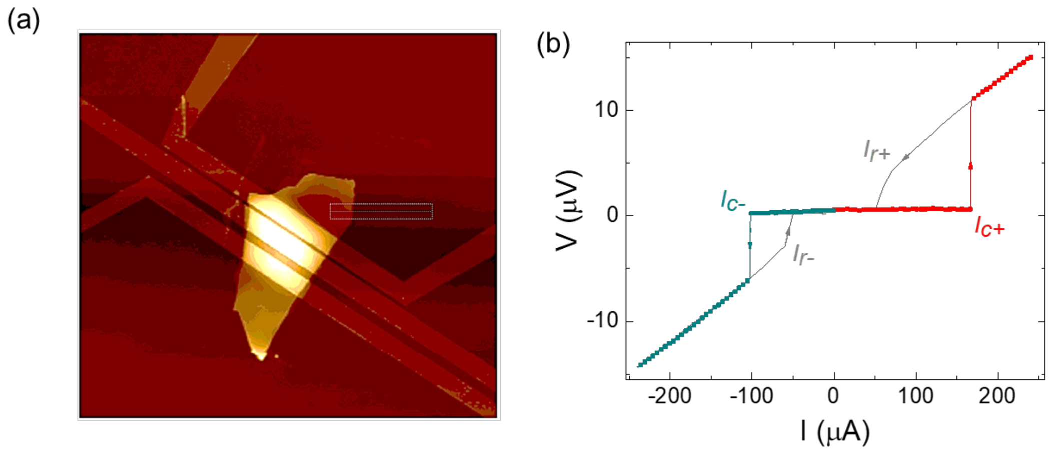

Fig. S5| Non-reciprocal effect in a 60 nm thick NiTe₂ Josephson junction. (a) Optical microscopy image of several JJ devices formed on a single $NiTe_2$ exfoliated flake with a thickness of ~ 60 nm. (b) $I - V$ curve of the JJ device with d = 350 nm in the presence of an in-plane magnetic field $B_y = 40$ mT that shows a large non-reciprocal critical current, I_{c-} and I_{c+}.

IV. Multiple sign reversals in ΔI_c as a function of in-plane magnetic field

Fig. S6 shows evidence of multiple sign reversals in ΔI_c when the magnitude of an in-plane magnetic field, B_y, is varied along a direction perpendicular to the current direction. In the main manuscript, only one sign-reversal is shown (up to ± 35 mT). From the phenomenological theory presented in the main text, each sign reversal (corresponding to $\Delta I_c = 0$ in Fig. S6) occurs when the phase shift is $n\pi$.

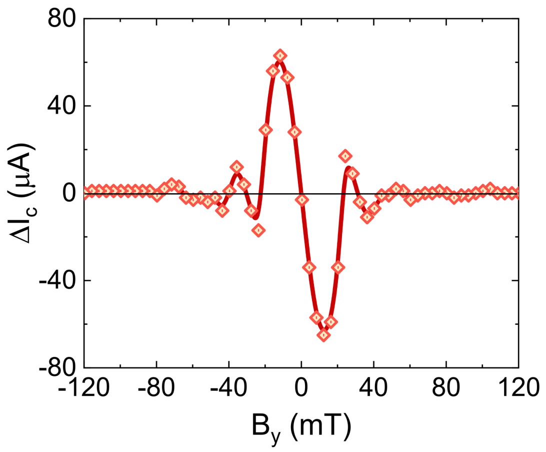

Fig. S6| Sign reversals of ΔI_c with applied magnetic field. The dependence of ΔI_c on B_y shows that the sign of ΔI_c reverses multiple times, which should take place whenever the phase shift equals an integer multiple of π (i.e. $n\pi$).

Dependence of sign reversal of ΔI_c on B_y at various temperatures

The color plot in Fig. S7 shows the variation of ΔI_c on B_y at various temperatures. The magnitude of ΔI_c oscillates as B_y is varied and decreases monotonically as the temperature is increased. This variation is explained in detail in the main manuscript.

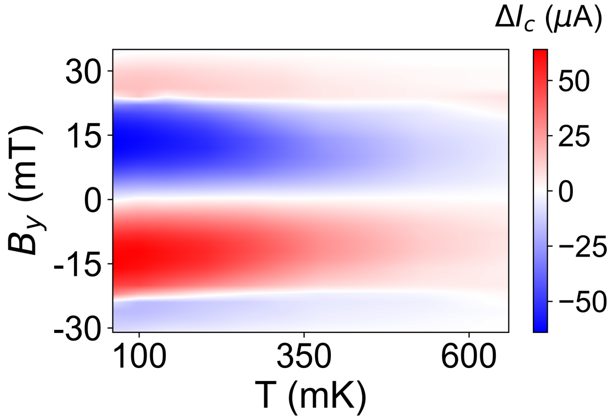

Fig. S7| Temperature and field dependent evolution of ΔI_c. The dependence of ΔI_c on B_y and temperature. The color code represents the magnitude of ΔI_c in units of μA as shown on the right hand color bar scale.

V. Determination of B_c and B_d for the theoretical model

When the magnetic field is applied along the y-direction, the dependence of the nonreciprocal part of the critical current is given by:

$$\Delta I_c \propto \Delta^4 \sin\delta \propto \Delta(B_y)^4 \sin\left(\pi \frac{B_y}{B_d}\right) \tag{S1}$$

Thus, the sign reversal is achieved whenever $B_y = nB_d$. Therefore, we estimate $B_d \approx 22$ mT from the position of the first sign change in the experiment.

In the main text of the paper, we concentrate on small values of magnetic fields and use the conventional dependence of the order parameters on the magnetic field $\Delta_{1,2} \propto \sqrt{1-\left(\frac{|\boldsymbol{B}|}{B_c}\right)^2}$ in order to find the qualitative behavior of the JDE effect with the magnitude of the in-plane field.

To find B_c, we compare the ratio of the magnitudes of the first and second maxima of the field dependence of ΔI_c to the same ratio extracted from the experiments. This yields $B_c \approx 45$ mT and the corresponding dependence of ΔI_c on the magnitude of the magnetic field in the y-direction is shown in Fig. S8.

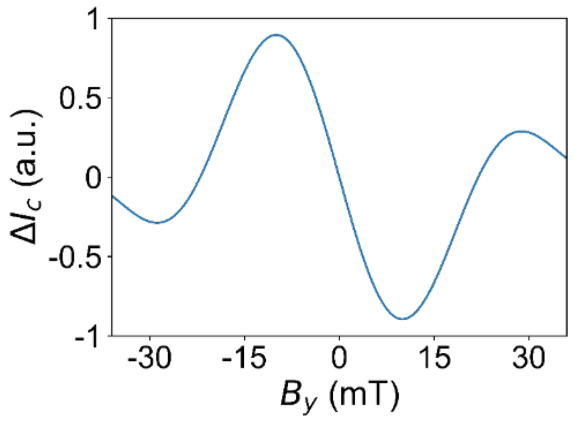

Fig. S8| Variation of ΔI_c with applied magnetic field from theory. Dependence of ΔI_c on B_y under the assumption $\Delta_{1,2} \propto \sqrt{1-\left(\frac{|B|}{B_c}\right)^2}$. The curve corresponds to $B_d = 22$ mT and B_c = 45 mT.

VI. Tilting correction and slope determination from the interference pattern to estimate finite momentum Cooper pairing

Previous reports [4,5] show that the dependence of the differential resistance $\frac{dV}{dI}$ as a function of B_z and B_x can provide insight into the finite momentum Cooper pairing. The variation of the interference pattern ($\frac{dV}{dI}$ vs B_z) under an in-plane magnetic field parallel to the current (B_x) closely resembles the oscillatory behavior of the Fraunhofer pattern[4,5]. With an increase in B_x, the interference pattern shifts along the B_z direction and the relative critical current of the side lobes increases as the critical current of the central lobe vanishes. To measure the evolution of the interference pattern ($\frac{dV}{dI}$ vs B_z), we apply an AC excitation (without any dc current) and measure the differential resistance $\frac{dV}{dI}$ as a function of B_z and B_x. We note that a lower

differential resistance corresponds to a higher critical current in the conventional Fraunhofer pattern[4,5].

Fig. S9a shows the dependence of $\frac{dV}{dI}$ on B_z and B_x. As discussed in the previous section, an application of B_x in the direction of the supercurrent flow causes the interference pattern to shift in a certain direction. To remove this shift/tilt, we estimate the slope of the tilt to be ~ 1/65 and the resulted $\frac{dV}{dI}$ map after slope correction is shown in Fig. S9b. The tilting is performed so that the central lobe is oriented vertically to allow for the calculation of the angles of the side branches.

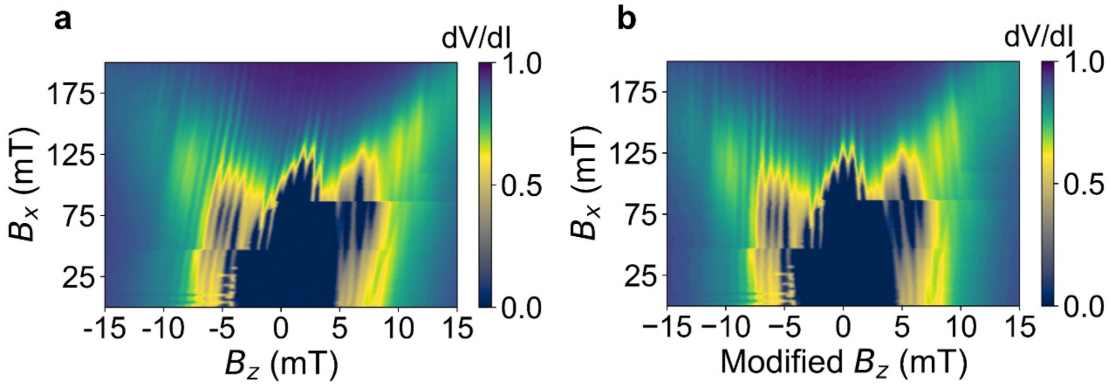

Fig. S9| Finite momentum cooper pairing in a d = 350 nm Josephson device. a, Tilted interference pattern highlighting the diminishing intensity of the central lobe on the application of a small in-plane magnetic field along the supercurrent flow. **b,** Corresponding rotated interference pattern for facilitating the calculation of the slope of the emerging side branches.

For a quantitative estimate of the finite momentum pairing, we calculate the ratio $\frac{B_x}{B_z}$ from the experiment (Fig. S10a). For several specific values of B_z (Fig. S10a), the dependence of $\frac{dV}{dI}$ on B_x is plotted, as shown for B_z = -13.46 mT and -9.83 mT in Fig. S10b and c, respectively. Using a polynomial fit, we calculate the value of B_x which corresponds to the minimum value of $\frac{dV}{dI}$. From these data, we calculate $\frac{B_x}{B_z}$ for positive and negative B_z separately (Fig. S10d): these ratios are ~15 and ~11, respectively. Thus, we use an average value of 13 for our calculations.

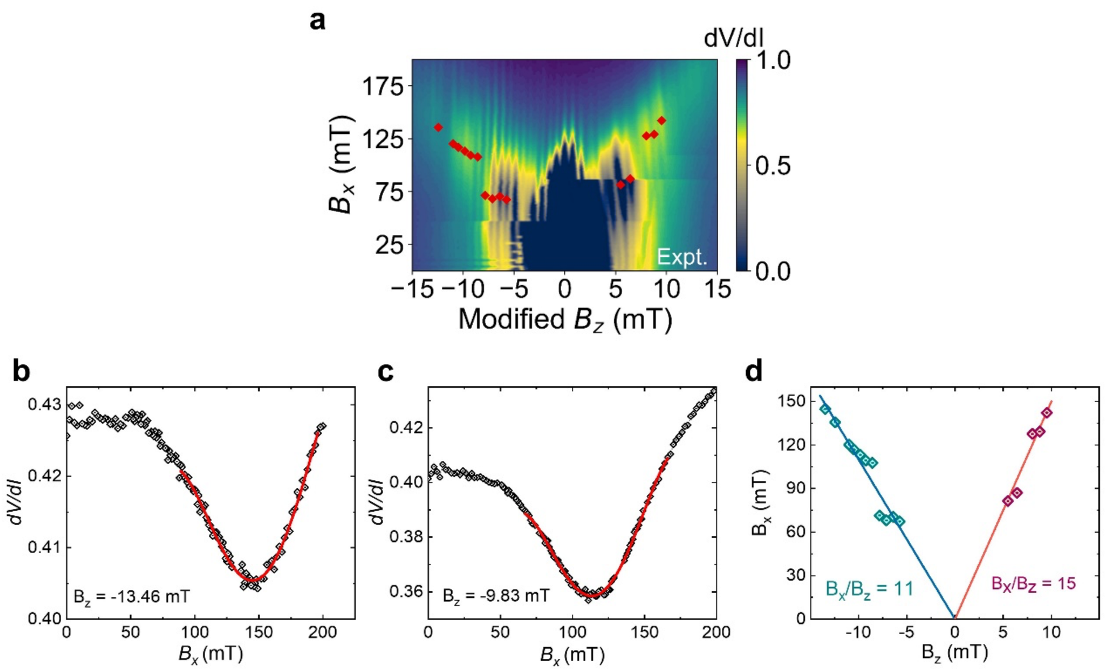

Fig. S10| Quantification of finite momentum cooper pairing. a, Dependence of $\frac{dV}{dI}$ on B_z and B_x (this figure is the same as Fig. 3b). **b,** and **c,** Dependence of $\frac{dV}{dI}$ on B_x for two specific values of B_z. A polynomial is used to fit the data (red solid line) and to calculate B_x that corresponds to the minimum value of $\frac{dV}{dI}$. **d,** Dependence of B_x on B_z is fitted to calculate the slopes separately.

Finite momentum pairing in d = 580 nm JJ device

Fig. S11 shows the evolution of the $\frac{dV}{dI}$ under B_z and B_x for d = 580 nm device. Similar to the d = 350 nm device, one can see that with increase in B_x the intensity of the central lobe is suppressed and divergent side branches branching are emerging in both sides of the diminished central lobe.

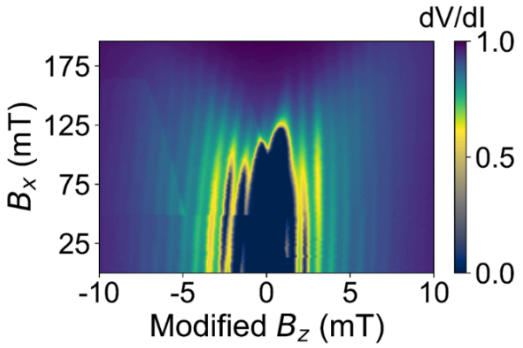

Fig. S11| $\frac{dV}{dI}$ as a function of B_z and B_x for the d = 580 nm device. In-plane field dependent evolution of the differential resistance shows the presence of finite momentum pairing for the d = 580 nm device.

VII. Calculation of the evolution of the Fraunhofer pattern

We calculate the Josephson current by summing quasi-classical trajectories of Cooper pairs across the junction (Fig. S12)[4]:

$$I(\Delta\varphi_0, B_x, B_z) = \int_0^W \int_0^W dy_1 dy_2 \frac{1}{d_{eff}^2 + (y_2 - y_1)^2} \sin\big(\Delta\varphi(B_x, B_z)\big) \quad \text{(S2)}$$

where,

$$\Delta\varphi(B_x, B_z) = \Delta\varphi_0 + \frac{2\pi B_z d_{eff}(y_1 + y_2)}{\Phi_0} + 2q_y(B_x)(y_2 - y_1) \quad \text{(S3)}$$

is the total phase difference for a trajectory that starts at $(0, y_1)$ and ends at (d_{eff}, y_2) , and $d_{eff} = d + 2\lambda$, $\lambda =$ 140 nm is the London penetration depth[6]. $\Delta\varphi_0$ is the phase difference between the order parameters in the two superconducting leads in the absence of applied field, and we have neglected the effect of the finite thickness of $NiTe_2$ (see the next section for a justification).

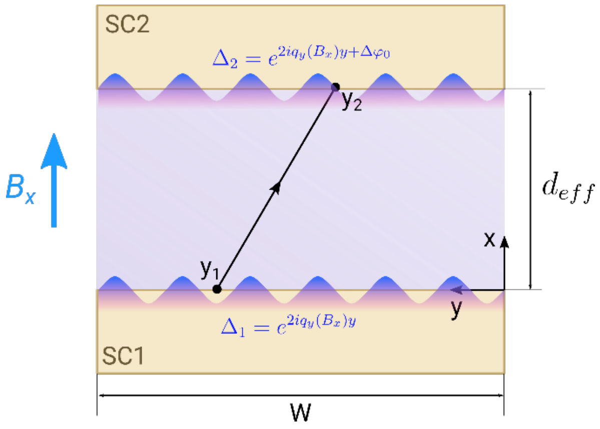

Fig. S12| Cooper pair tunneling across a Josephson junction. Schematic diagram illustrating Cooper pair tunneling from point $(0, y_1)$ to (d_{eff}, y_2) between two superconductors with a Cooper pair momentum $2q_y(B_x)$.

In order to calculate the evolution of the Fraunhofer pattern, we compute the Josephson current using eq. (S2) and maximize it by varying $\Delta\varphi_0$, which allows us to find I_c. To compare theoretical and experimental predictions, we further adjust the value of the effective junction separation d_{eff} because of flux focusing[4], which is carried out by calculating the Fraunhofer pattern at zero in-plane magnetic field using eq. (S2) (which gives a slightly different, but still

qualitatively similar dependence to $\frac{Sin(\frac{\pi\Phi}{\Phi_0})}{(\frac{\pi\Phi}{\Phi_0})}$ and is shown in Fig. S13 below) and fit the position of the first minimum to the experimental value from the experiments shown in Fig. S8. As we see in Fig. S13, the central peak disappears, and two side branches emerge as the value of the magnetic field is increased. We find the linear dependence of the parameter $q(B_x)$ in eq. (S3) and the vertical scale of the theoretical plot by using the average slope of the side branches $\frac{B_x}{B_z}$ from experiment and matching it to the slope that the calculated pattern has[4]: $\frac{2q_y}{B_z} \approx \frac{\pi d_{eff}}{\Phi_0}$.

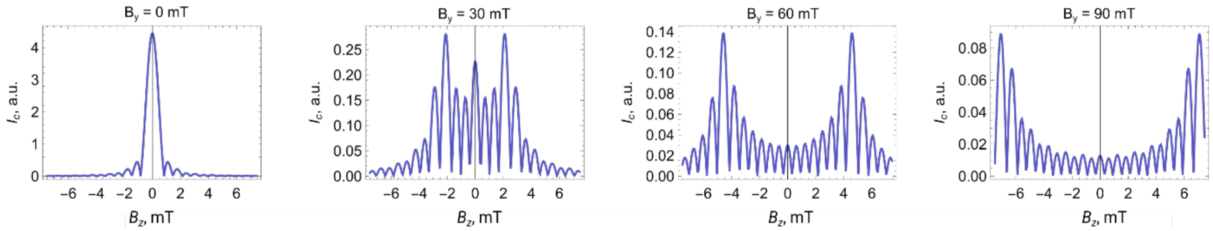

Fig. S13| Theoretical calculation of Fraunhofer pattern evolution with magnetic field. Fraunhofer patterns at several values of in-plane magnetic field as computed from maximizing eq. (S2).

VIII. Effect of the finite film thickness of $NiTe_2$ on the evolution of the Fraunhofer pattern

When the thickness t is finite, there will be another contribution to the phase in eq. (S3) from the flux piercing the device through the y-z plane.

$$\Delta\varphi_t(B_x) = \frac{B_x(y_2 - y_1)t}{\Phi_0} \tag{S4}$$

Let us compare the maximum contribution of this phase in comparison to the other two in (S3). For example, let us compare $\Delta\varphi_t(B_x)$ to the phase due to the Cooper pair momentum $2q_y(B_x)(y_2 - y_1)$ at typical values of fields for the Fraunhofer map: $B_x \approx 100$ mT and $B_z \approx 10$ mT:

$$\frac{\Delta\varphi_t(B_x)}{2q_y(B_x)(y_2 - y_1)} = \frac{B_x t}{2\Phi_0 q_y(B_x)} \tag{S5}$$

As discussed above, we can estimate the Cooper pair momentum from $2q_y \approx \frac{\pi d_{eff}}{\Phi_0} B_z$, which yields:

$$\frac{\Delta\varphi_t(B_x)}{2q_y(B_x)(y_2 - y_1)} = \frac{1}{2\pi} \frac{t}{d_{eff}} \frac{B_x}{B_z} \sim 0.06 \tag{S6}$$

We have used a typical thickness of 20 nm and d_{eff} ~1 μm. Therefore, we can neglect the contribution from the finite thickness effect in comparison to the phase due to the finite Cooper pair momentum.

Additionally, we estimate the flux piercing through the cross-section of the device perpendicular to the y-axis (x-axis is parallel to the current), corresponding to maximum dimension 20nm x 350nm, is $\frac{\Phi}{\Phi_0} \approx 0.06$. When the field is turned by 90 degrees, the probabilities of trajectories with finite transversal shift $y_2 - y_1$ that would be pierced by non-zero flux are suppressed by the factor $\left(\frac{d^2}{d^2+(y_2-y_1)^2}\right)$ (see ref. [5]), and the average flux is this case is similarly small.

IX. ARPES data and Fermi velocity of two surface states

Previous reports[7,8] show that $NiTe_2$ hosts spin-split topological surface states (SS) along with a bulk type-II Dirac semi-metallic ground state. Our theoretical calculations suggest that both the low Fermi velocity and small Fermi energy of one of these SSs play key roles in creating the JDE (see main text). In order to extract these material parameters, angle-resolved photoelectron spectroscopy (ARPES) experiments were carried out on a $NiTe_2$ single-crystal cleaved along the [0001] direction. The energy-momentum dispersion along the $\overline{\Gamma} \rightarrow \overline{M}$ direction is given in Fig. S14a, where two topological surface states (SS1 and SS2) are indicated with arrows. These experimental findings can be well reproduced by our *ab-initio* calculations (Fig. S14b), which also indicate the spin-polarization of SS1 and SS2.

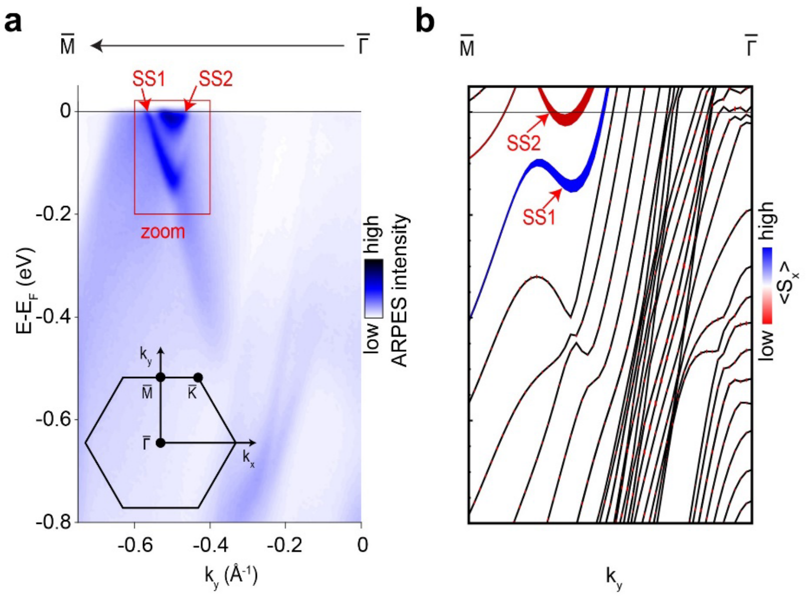

Fig. S14| ARPES data on $NiTe_2$. a, Energy momentum dispersion along the $\bar{\Gamma} \rightarrow \bar{M}$ direction where two distinct topological surface states (SS1, SS2) cross the Fermi level. **b,** Spin polarized ab-initio calculations show good agreement with our experimental data.

To calculate the Fermi velocities of both SS1 and SS2, we extract several energy distribution curves (EDCs) from Fig. S14a. Fig. S15a shows selected EDCs in the k_y range from -0.542 $Å^{-1}$ to -0.442 $Å^{-1}$. Each EDC was fitted using a spline function to extract the peak positions of both the SS1 and SS2 states. Fig. S15b and c show the dependence of the peak position as a function of k_y for SS1 and SS2, respectively. The peak positions close to the Fermi energy (E_F) were fitted with a linear function to calculate the slope, $\frac{dE}{dK}$, for both surface sates, which are close to - 2.2 eV.Å and -0.26 eV.Å for SS1 and SS2, respectively. Form these slopes we calculated the Fermi velocities of both surface states using $v_F = \frac{1}{\hbar}\frac{\partial E}{\partial k}$, which are $3.3 \times 10^{+5}$ m/s and $0.4 \times 10^{+5}$ m/s for SS1 and SS2, respectively.

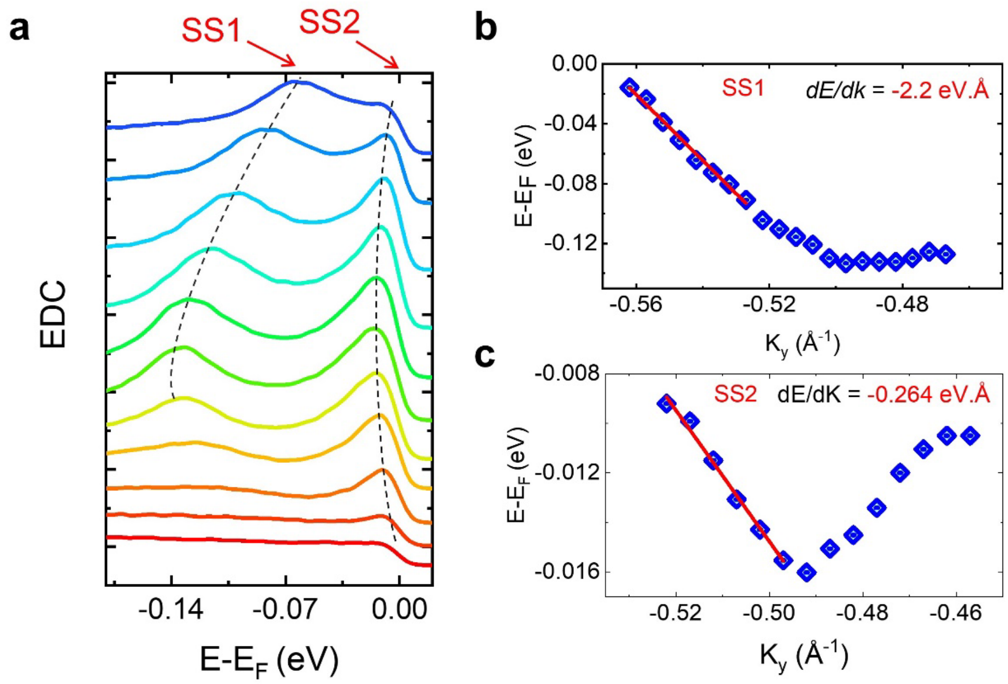

Fig. S15| Fermi velocity calculation from the ARPES data. a, Energy Distribution Curve (EDC) obtained from figure S14a in the k_y range from -0.542 $Å^{-1}$ to -0.442 $Å^{-1}$ with the gap between the two curves Δk_y ~ -0.01 $Å^{-1}$. Each curve was fitted with a spline function to calculate the peak position. **b-c,** Dependence of the peak position as a function of k_y are fitted with a linear function to calculate $\frac{dE}{dk}$ for SS1 and SS2, respectively.

X. Photon energy dependent ARPES measurements for the identification of the surface states:

We identify the existence of the topological surface states by carrying out photon energy dependent ARPES measurements, as shown in Fig. S16. One can see that the bands identified as e-SS and h-SS in Fig. S16 have negligible out-of-plane dispersion (photon energy dependent dispersion), which is the hallmark of a state confined to the 2D surface of the crystal. This is in line with previous investigations in the literature[7,8].

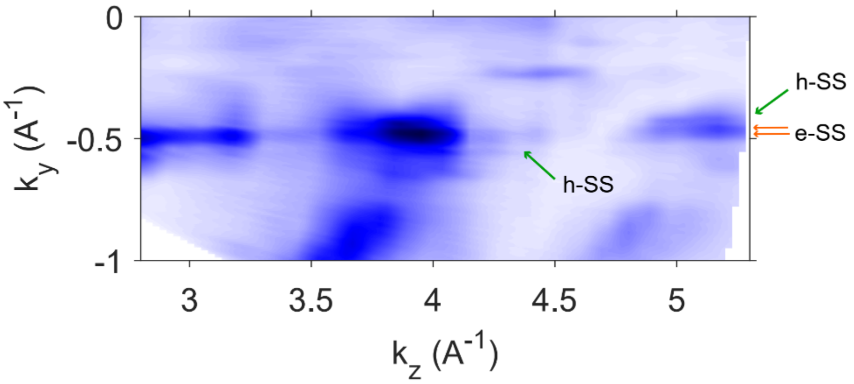

Fig. S16| Photon energy dependent ARPES measurements: Photon energy dependent Fermi surface map of $NiTe_2$ showing the electron-like surface state (e-SS) and hole-like surface states (h-SS), as indicated in the figure. These states are not dispersive along the k_z direction.

References:

1 Liu, Q. *et al.* Nontopological origin of the planar Hall effect in the type-II Dirac semimetal $NiTe_2$. *Phys. Rev. B* **99**, 155119 (2019).

2 Tinkham, M. Introduction to Superconductivity. *Dover Publication Inc* (2004).

3 Barone, A. & Paterno, G. Physics and Applications of the Josephson Effect, Second Edition. *John Wiley & Sons* (1982).

4 Chen, A. Q. *et al.* Finite momentum Cooper pairing in three-dimensional topological insulator Josephson junctions. *Nat. Commun.* **9**, 3478 (2018).

5 Hart, S. *et al.* Controlled finite momentum pairing and spatially varying order parameter in proximitized HgTe quantum wells. *Nat. Phys.* **13**, 87-93 (2017).

6 Gubin, A. I., Il'in, K. S., Vitusevich, S. A., Siegel, M. & Klein, N. Dependence of magnetic penetration depth on the thickness of superconducting Nb thin films. *Phys. Rev. B* **72**, 064503 (2005).

7 Ghosh, B. *et al.* Observation of bulk states and spin-polarized topological surface states in transition metal dichalcogenide Dirac semimetal candidate $NiTe_2$. *Phys. Rev. B* **100**, 195134 (2019).

8 Mukherjee, S. *et al.* Fermi-crossing Type-II Dirac fermions and topological surface states in $NiTe_2$. *Sci. Rep.* **10**, 12957 (2020).
